# A Robust Tensor-Based Submodule Clustering for Imaging Data Using $l_{\frac{1}{2}}$ Regularization and Simultaneous Noise Recovery via Sparse and Low Rank Decomposition Approach

**DOI:** 10.3390/jimaging7120279

**Published:** 2021-12-17

**Authors:** Jobin Francis, Baburaj Madathil, Sudhish N. George, Sony George

**Affiliations:** 1 Department of Electronics and Communication Engineering, National Institute of Technology Calicut, Calicut 673601, India; jobinkapyarumalayil@gmail.com (J.F.); sudhish@nitc.ac.in (S.N.G.); 2 Department of Electronics and Instrumentation, Government Engineering College Kozhikode, Calicut 673005, India; baburajmadathil@gmail.com; 3 Department of Computer Science, Norwegian University of Science and Technology, 2815 Gjøvik, Norway

**Keywords:** subspace clustering, submodule clustering, $l_{\frac{1}{2}}$ induced tensor nuclear norm (TNN), sparse and low rank decomposition

## Abstract

The massive generation of data, which includes images and videos, has made data management, analysis, information extraction difficult in recent years. To gather relevant information, this large amount of data needs to be grouped. Real-life data may be noise corrupted during data collection or transmission, and the majority of them are unlabeled, allowing for the use of robust unsupervised clustering techniques. Traditional clustering techniques, which vectorize the images, are unable to keep the geometrical structure of the images. Hence, a robust tensor-based submodule clustering method based on $l_{\frac{1}{2}}$ regularization with improved clustering capability is formulated. The $l_{\frac{1}{2}}$ induced tensor nuclear norm (TNN), integrated into the proposed method, offers better low rankness while retaining the self-expressiveness property of submodules. Unlike existing methods, the proposed method employs a simultaneous noise removal technique by twisting the lateral image slices of the input data tensor into frontal slices and eliminates the noise content in each image, using the principles of the sparse and low rank decomposition technique. Experiments are carried out over three datasets with varying amounts of sparse, Gaussian and salt and pepper noise. The experimental results demonstrate the superior performance of the proposed method over the existing state-of-the-art methods.

## 1. Introduction

Classification of data into sensible groups is essential in a wide variety of fields, such as engineering, medical science, business, marketing and many more [1,2]. The most popular approaches for classifying objects into groups are discriminant analysis and clustering techniques [2,3]. Discriminant analysis is a supervised learning method in which the class labels are already defined and the aim is to find the data that have not been labeled [2,4]. In clustering, the problem is to group the unlabeled data into sensible groups. Hence, clustering is useful in applications where there is little prior information about the available data [3]. Due to massive data generation in recent years, clustering has been found useful in various fields such as machine learning, pattern analysis, decision making, etc. [5]. The popular clustering algorithms proposed in recent years include hierarchical clustering, partitioning clustering, mixture resolve clustering, fuzzy clustering, and so on [2,6]. The methods described above take into account all of the dimensions of the input data during learning. Dealing with high-dimensional datasets, on the other hand, can be more difficult due to the curse of the dimensionality problem [7,8]. As the dimensionality of the data grows, the data can become sparser, increasing the computational complexity of clustering [5].

Even if the data are multidimensional, they can be expressed effectively in a union of low-dimensional space [9]. In real-world scenarios, the high-dimensional data would also be distributed across several low-dimensional subspaces [10,11]. Then, the aim of subspace clustering is to identify these subspaces and segment the data based on their dissimilarity [7,12]. Algebraic methods, matrix factorization methods, statistical methods, and spectral clustering methods are the major types of subspace clustering techniques [6,13,14]. Spectral clustering is simple to implement and can outperform traditional algorithms. Hence, it is the most popular method for high-dimensional data clustering [15]. Depending on the type of affinity matrices derived from the data, various spectral clustering algorithms have been proposed.

Shi et al. proposed a normalized spectral clustering method which measures the dissimilarity between different groups and the similarity within the group using a normalized Laplacian matrix [16]. Andrew et al. proposed another method with additional row normalization [17]. Then, the sparse subspace clustering (SSC) algorithm proposed by Elhamifar et al. utilizes the self-expressiveness property of the data [10]. The underlying theory behind the self-expressiveness property is that every data point lying in a particular subspace can be expressed as a linear combination of other data points that belong to the same subspace [1,10]. The SSC algorithm aims to find a sparse representation that corresponds to a minimal set of points belonging to the same subspace. Then, the solution of the optimization problem is used for spectral clustering [10]. Liu et al. proposed subspace segmentation by low rank representation (LRR) [18]. Similar to SSC, LRR also represents a given data point as the linear combination of other data points [14] but instead of sparsest representation, LRR tries to find the low rank representation.

When dealing with higher dimensional signals, such as images, all the aforementioned methods map the 2D images into one-dimensional vectors. This approach is not so effective in capturing the spatial structure information of the images. To address this problem, instead of vectorizing the imaging data, a new approach called the union of free submodule (UoFS) model was proposed, which preserves the spatial structure of the 2D data [18,19]. In this model, images are stacked together in a third order tensor space. Kernfeld et al. proposed sparse submodule clustering (SSmC), which combines the UoFS model with the self-expressiveness property exploited in the SSC algorithm. In this, each image is interpreted as a linear combination of remaining images in the dataset [20]. However, in SSmC, the correlation between images from the same submodule is not taken into account [21]. To consider the inner correlation, the low rank structure of the multi-linear data is exploited in the sparse and low-rank submodule clustering method (SLRSmC) proposed by Piao et al. [21]. Identical to the scalar product, the tensor product is utilized for constructing the submodule clustering method. SLRSmC, on the other hand, imposes a low rank constraint on each image in the tensor, rather than a tensor low rank constraint. Wu et al. resolved this problem by imposing a low tensor rank constraint using the tensor nuclear norm (TNN) [19].

For enforcing the low rank constraint, the methods proposed in [19,20,21] use $l_{1}$ norm instead of $l_{0}$ norm. This ensures that the optimization problem is convex since $l_{1}$ norm is considered the convex surrogate of $l_{0}$ norm [22]. Relying on the UoFS model, many extensions of the work proposed by Wu et al. were developed with the objective of addressing real-world scenarios, such as noise, incomplete observations, and so on. Francis et al. proposed a tensor-based single stage optimization framework for clustering imaging data under incomplete observations [6]. In this work, individual images with missing samples are fetched in sequence from the input data tensor for reconstruction. Further, reconstruction of the missing samples is carried out by the matrix completion [6]. In another work, Johnson et al. replaced the low tensor multirank equivalent TNN by employing weighted tensor nuclear norm minimization (WTNN) for a more accurate low rank representation [23]. Baburaj et al. proposed a noise robust tensor-based submodule identification approach, named re-weighted low rank tensor approximation and $l_{\frac{1}{2}}$ regularization $\left(\mathrm{RLRTA}l_{\frac{1}{2}}R\right)$ to perform clustering in the presence of gross errors [24], using the re-weighted tensor nuclear norm. An error term was introduced into the model to separate noise and data, which brings noise robustness to the clustering technique. Xia et al. proposed a subspace clustering method for multi-view data in which the representation tensor is learned by means of weighted tensor Schatten *p*-norm minimization (WTSNM) [25]. In another work, Wu proposed a clustering-aware Laplacian regularized low-rank submodule clustering (CLLRSmC) model that exploits the local manifold structure of the data [26]. In this work, the nonlinear extension of the UoFS model which can adapt data drawn from a mixture of nonlinear manifolds was presented.

Concurrently, the principle of sparse and low rank decomposition of matrices and tensors was applied to many research problems for noise removal. Shijila et al. proposed a unified framework of simultaneous denoising and moving object detection using low rank approximation [27]. Jin et al. proposed an impulse noise removal algorithm named robust ALOHA, which employs a sparse and low rank decomposition of a Hankel structured matrix [28]. They modeled impulse noise as a sparse component, then restored the underlying image while preserving the original image features [28]. Similarly, Cao et al. proposed a subspace-based non-local low rank and sparse factorization (SNLRSF) method for hyperspectral image denoising [29].

Since real-world data are heavily influenced by noise, which reduces clustering efficiency, and current techniques are unable to completely recover the data from noise, we propose a robust tensor based submodule identification technique with improved clustering capability, taking the following factors into account.

1. A robust tensor-based submodule clustering algorithm is proposed in this paper, which combines the clustering of 2D images with simultaneous noise removal in a single framework. Real-world data, such as images and videos, are frequently subjected to noise during acquisition, transmission, or due to limitations imposed by material and technological resources. The presence of noise affects the performance of clustering algorithms. To limit the effects of noise, existing methods usually include a global error term in their optimization problem. However, following this approach will not fully remove the noise encountered in individual images.
2. Hence, this work proposes a simultaneous noise removal scheme based on twisting the third-order input data tensor, which allows lateral image slices to become frontal slices of the twisted data tensor. Furthermore, images are extracted from this tensor data one by one, and each image is subjected to a sparse and low rank decomposition approach. Unlike the existing clustering methods, this procedure can find and eliminate the noise content in each of the images from the data, and a clean noise-free data tensor can be obtained for further clustering.
3. To better capture the low rankness and self-expressiveness property, $l_{\frac{1}{2}}$ induced TNN is integrated into the proposed method. Furthermore, $l_{\frac{1}{2}}$ regularization is incorporated into the submodule identification term because of its ability to induce more sparsity. An optimization problem is formulated that enables the proposed method to perform improved clustering, even in the presence of noise by employing the capabilities of $l_{\frac{1}{2}}$ induced TNN and $l_{\frac{1}{2}}$ regularization, as well as simultaneous noise removal using sparse and low rank decomposition.

## 2. Technical Background

In this paper, tensors, matrices, vectors and scalars are denoted by calligraphic uppercase, bold uppercase, bold lowercase and nonbold letters, respectively. For a third-order tensor X, X(:,l,m),X(l,:,m) and X(l,m,:) represent the ${\left(l,m\right)}^{th}$ mode-1, mode-2 and mode-3 (tube) fibers respectively [19]. Similarly, X(i,:,:),X(:,i,:) and X(:,:,i) denote the $i^{th}$ horizontal, lateral and frontal slices, respectively. Then, the $i^{th}$ frontal slice can also be represented by $X^{\left(i\right)}$. Finally, $x_{l,m,n}$ represents the ${\left(l,m,n\right)}^{th}$ element of X. The rest of this paper is organized as follows. Section 2 presents the technical background. The proposed optimization model and its solutions are described in Section 3. In Section 4, the performance of the proposed method is evaluated and the experimental results are presented. The conclusions are drawn in Section 5.

### 2.1. Sparse and Low Rank Matrix Decomposition

It is evident that natural images have a low rank structure and those of the same rank will be increased when they are affected by noise [30]. Hence, noise removal can be done by decomposing the data into a low rank and sparse component. Selecting the low rank part only would provide noise-free data. In the sparse and low rank decomposition method, a given matrix, X can be expressed as the sum of low rank component T and sparse component S [22,31]. The formulation is given by [27]

(1) $$\begin{array}{cc} \;\;\;\;\;\;\;\;\;\;\;\;\;\min_{T,S}\; & \mathrm{rank}\left(T\right)+\lambda {∥S∥}_{0}\\ \; & s.\;t.\;X=T+S \end{array}$$

where, T denotes the low rank matrix, S represents the sparse matrix and λ represents the regularization term. However, solving Equation (1) is NP hard due to its non-convex nature [27]. In principal component pursuit (PCP), Candes et al. recovered the low rank matrix by convex programming tools by the following formulation,

(2) $$\begin{array}{cc} \;\;\;\;\;\;\;\;\;\;\;\;\;\;\;\min_{T,S}\; & {∥T∥}_{*}+\lambda {∥S∥}_{1}\\ \; & s.\;t.\;X=T+S \end{array}$$

where ${∥.∥}_{*}$ represents the nuclear norm and ${∥.∥}_{1}$ represents the $l_{1}$ norm. The nuclear norm of a matrix T is given by the absolute sum of singular values, and the minimization of ${∥T∥}_{*}$ imposes a low rank nature [27]. The singular value decomposition (SVD) [32] approach with a specific threshold can be applied to obtain the low rank in which singular values are arranged in descending order. Since the first few singular values hold maximum energy, smaller singular values can be omitted, as those values usually represent noise or other sparse corruptions [27,33,34]. However, solving Equation (2) reduces the sparse content alone, and hence, mitigating factors, such as Gaussian noise or group sparsity, cannot be taken into account [22]. Zhou et al. extended the problem in Equation (2) by adding an equality constraint, ${∥X-T-S∥}_{F}^{2}\leq \sigma$, into their model in order to handle Gaussian noise and other arbitrary corruptions, where σ is the threshold [22]. The formulation is given by [22]

(3) $$\begin{array}{cc} \;\;\;\;\;\;\;\;\;\;\min_{T,S}\; & {∥T∥}_{*}+\lambda {∥S∥}_{1}+\frac{\mu }{2}{∥X-T-S∥}_{F}^{2} \end{array}$$

where μ represents the penalty parameter [6,22].

### 2.2. Self-Expressiveness Property of Submodules

Since images and videos in real life often have noise components associated with them, the management and grouping has become more challenging. The basic problem of image clustering is to group a collection of *N* images $Y={\left\{Y_{i}\in R^{n_{1}\times n_{3}}\right\}}_{i=1}^{N}$ into *L* categories [19]. However, the majority of existing clustering methods consider the images as vectors belonging to an $n_{1}n_{3}$-dimensional space. Although this is a reasonable approach in many cases, in image clustering, where the geometrical structure of the data have to be taken into account, it cannot provide satisfactory results. In the UoFS model, the images are considered to be lateral slices of a tensor $Y\in R^{n_{1}\times N\times n_{3}}$[19]. The images can be assumed to belong to a union of free submodules. Then, the problem of finding the clusters is equivalent to finding out the submodules to which each image belongs. This approach takes into account the spatial aspects of the images. Let $K_{n_{3}}$ denote the set of all tubes belonging to $R^{1\times 1\times n_{3}}$. The above set of tubes can then be used to form a commutative ring under regular addition and *t*-product [19,35]. The set of $n_{1}\times 1\times n_{3}$ lateral slices can be denoted by $K_{n_{3}}^{n_{1}}$. Similar to vector spaces over field, $K_{n_{3}}^{n_{1}}$ forms a free module over the ring $K_{n_{3}}$. As a result, a free submodule over the ring can be thought of as a generalized version of the subspace over the field [6,19].

## 3. Proposed Method

**Figure 1:**
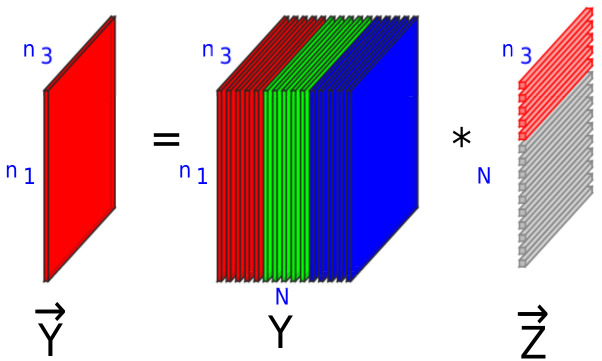
Self-expressiveness property of free submodules. Red fibers represent non-zero fibers and greyish fibers represent zero value fibers. Non-zero fibers represent coefficients from intra-cluster. Zero fibers denote coefficients from inter-clusters.

Under the UoFS model, making use of the self-expressiveness property, an image fetched from a submodule can be expressed as the *t*-linear combination of other images present in the same submodule, as shown in Figure 1. Hence, for a third-order tensor $Y\in R^{n_{1}\times N\times n_{3}}$, there exists a coefficient tensor, $Z\in R^{N\times N\times n_{3}}$ such that Y≈Y*Z[6]. Further, the tensor multirank can be used to capture the self-expressiveness property [19] using the tensor nuclear norm ${∥.∥}_{⊛}$, which is the tightest convex relaxation of the tensor multirank [36]. Since the structure of the solution Z also determines the performance of the clustering, a block diagonal structure is required for the coefficient tensor, Z, which can reveal the compactness between the intraclass components and the separation between the interclass components [8,37,38]. Hence, in the UoFS model, an f-block diagonal structure constraint is added [19]. In addition, images which belong to a single submodule are highly correlated, while those that belong to different submodules are slightly correlated; to capture this, a dissimilarity matrix, $M\in {\left[0,1\right]}^{N\times N}$ is defined, where each entry indicates the dissimilarity between two images. The entries of M, $m_{k,l}$ are given by [19]

(4) $$\;\;\;\;\;\;m_{k,l}=1-exp\left(-\frac{1-\left|\langle Y(:,k,:),Y(:,l,:)\rangle \right|}{\gamma }\right)$$

Here, Y(:,k,:) and Y(:,l,:) represent the $k^{th}$ and $l^{th}$ lateral slices of the 3rd order tensor Y, and γ is the empirical average of all $\left(1-\left|\langle Y(:,k,:),Y(:,l,:)\rangle \right|\right)$[19]. Once an optimum coefficient tensor Z, is obtained, the clustering of data can be achieved using the spectral clustering technique in which the ${\left(k,l\right)}^{th}$ entry of affinity matrix A can be calculated as [17]

(5) $$\;\;\;\;\;\;\;\;\;a_{k,l}={∥Z\left(k,l,:\right)∥}_{F}+{∥Z\left(l,k,:\right)∥}_{F}$$

Incorporating all the factors mentioned above, the clustering problem is formulated into an optimization problem given by [19]

(6) $$\min_{Z}\;{∥Z∥}_{⊛}+\lambda_{1}\sum_{k=1}^{n_{3}}{∥M⊙Z^{\left(k\right)}∥}_{1}+\lambda_{2}{∥Y-Y*Z∥}_{F}^{2}$$

where ${}^{‵}{⊙}^{\prime }$, ${∥.∥}_{1}$ and ${∥.∥}_{F}$ represent the element-wise multiplication operator, $l_{1}$ norm and Frobenius norm, respectively. Further, $∥Z{∥}_{⊛}$ represents the tensor nuclear norm (TNN) [19]. In addition, $\lambda_{1}$ and $\lambda_{2}$ stand for the regularization parameters for the optimization problem.

Further, $l_{1}$ norm is used in the submodule structure constraint term, $\lambda_{1}\sum_{k=1}^{n_{3}}{∥M⊙Z^{\left(k\right)}∥}_{1}$ as well as in TNN of Z in the above expression. The methods mentioned in [19,20,21], $l_{1}$ norm are used instead of $l_{0}$ norm for imposing the low rank constraint. The strong acceptance of $l_{1}$ minimization in sparsity-related problems is because of its convex nature and ability to provide the sparse solution with less computational bottleneck [39]. However, $l_{1}$ regularization is a loose approximation of $l_{0}$ regularization, and the performance will be limited in many applications [40,41]. Hence, to improve the performance, $l_{q}$ (0<q<1) regularization techniques can be employed. Therefore, in order to extract the sparsest structure for the vector $x\in R^{N}$, from the observation y=Ax, the $l_{q}$ regularization problem is represented by

(7) $$\;\;\;\;\;\;\;\;\;\;\;\;\;\;\min_{x\in R^{N}}{∥\mathrm{Ax}-y∥}_{2}^{2}+\lambda {∥x∥}_{q}^{q}$$

where $y\in R^{m}$, $A\in R^{m\times N}$. Then, ${∥x∥}_{q}$ represents the $l_{q}$ quasi-norm and is defined by, ${∥x∥}_{q}=(\sum_{i=1}^{N}|x_{i}{{|}^{q})}^{\frac{1}{q}}$.

**Figure 2:**
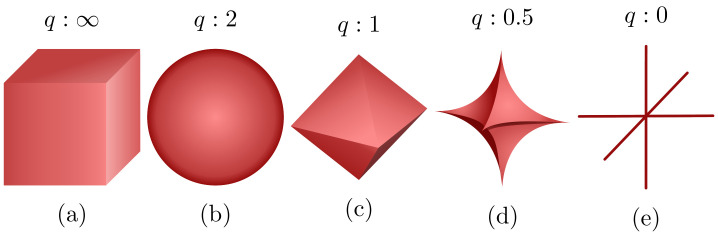
Unit ball representation of (**a**) $l_{\infty }$ norm (**b**) $l_{2}$ norm (**c**) $l_{1}$ norm (**d**) $l_{\frac{1}{2}}$ norm and (**e**) $l_{0}$ norm, in the three dimensional space $R^{3}$.

The unit ball representations of all the norms are illustrated in Figure 2 in which $l_{2}$ norm has the spherical shape, whereas in $l_{1}$ norm, it is diamond shaped. It is obvious that $l_{1}$ regularization provides a sparser solution compared to $l_{2}$ norm since there is higher probability for the y=Ax line to coincide with the axes. However, as the value of *q* is again reduced, the unit ball can assume the shape as shown in Figure 2d.

Hence, the probability of achieving sparser solution is higher as the value of *q* is changed from 0 to 1. For $q\in [\frac{1}{2},1)$, the solution will be sparser for a smaller value of *q*. No significant change is observed in the performance for $q\in [0,\frac{1}{2})$ [39,42,43]. Hence, $l_{\frac{1}{2}}$ regularization can be chosen as the optimum regularization method. In works such as [19,20,21], TNN was used for imposing the low rank constraint in their optimization problem. For a tensor $X\in R^{n_{1}\times n_{2}\times n_{3}}$, the expression for TNN with t-SVD $X=U*\Sigma *V^{T}$ is given by [19],

(8) $${\;\;\;\;\;\;\;\;∥X∥}_{⊛}=\sum_{k=1}^{n_{3}}\sum_{i=1}^{min(n_{1},n_{2})}\left|\hat{\Sigma }\left(i,i,k\right)\right|$$

where in $U\in R^{n_{1}\times n_{1}\times n_{3}}$ and $V\in R^{n_{2}\times n_{2}\times n_{3}}$ are the orthogonal tensors. In addition, $\Sigma \in R^{n_{1}\times n_{2}\times n_{3}}$ is an f-diagonal tensor and $\hat{\Sigma }$ is its Fourier transform. As in Equation (8), TNN uses the $l_{1}$ norm to determine the absolute sum of the singular values in each frontal slice of the tensor $\hat{\Sigma }$. However, in comparison to the $l_{1}$ norm, $l_{\frac{1}{2}}$ regularization yields a more sparse solution [39,43]. Hence, to obtain a more accurate low tensor rank representation, $l_{\frac{1}{2}}$ regularization is incorporated, and the Equation (8) can be rewritten as

(9) $${\;\;\;\;\;\;\;\;\;∥X∥}_{⊛\frac{1}{2}}=\sum_{k=1}^{n_{3}}\sum_{i=1}^{min(n_{1},n_{2})}\sqrt{|\hat{\Sigma }\left(i,i,k\right)|}$$

where the above expression can be called $l_{\frac{1}{2}}$ induced TNN. In TNN, the frontal slices of the tensor $\hat{\Sigma }$ contains $n_{3}$ frontal slices, each slice being a diagonal matrix with singular values $\sigma_{1}\geq \sigma_{2}...\geq \sigma_{n_{min}}\geq 0$, where $n_{min}=\min\left(n_{1},n_{2}\right)$ as entries. Then, we apply the half thresholding function proposed by Xu et al. over the vector $\sigma =(\sigma_{1},\sigma_{2},...,\sigma_{n_{min}})$, and it can be expressed as [39],

(10) $$\;\;h_{\lambda ,\frac{1}{2}}\left(\sigma_{i}\right)=\left\{\begin{array}{c} \frac{2}{3}\sigma_{i}\left(1+cos\left(\frac{2\pi }{3}-\frac{2}{3}\Psi_{\lambda }\left(\sigma_{i}\right)\right)\right),\\ \;\;\;\;\;\;\;\;\;\;\;\;\;\;\;\;\;\;\;\;\;\;\;\;\;\;\;\;\;\;\;\left|\sigma_{i}\right|>\frac{\sqrt[{3}]{54}}{4}{\left(\lambda \right)}^{\frac{2}{3}}\\ 0,\;\;\;\;\;\;\;\;\;\;\;\;\;\;\;\;\;\;\;\;\;\;\;\;\;\;\;\;\mathrm{otherwise} \end{array}\right.$$

where $\Psi_{\lambda }\left(\sigma_{i}\right)=\arccos\left(\frac{\lambda }{8}{\left(\frac{|\sigma_{i}|}{3}\right)}^{-\frac{3}{2}}\right)$ and i=1 to $n_{min}$. Then, using the non linear half thresholding operator, $H_{\lambda ,\frac{1}{2}}\left(.\right)$, we perform the expression given in Equation (10) for all elements of σ. The expression for the half thresholding operator $H_{\lambda ,\frac{1}{2}}\left(.\right)$ is given by $H_{\lambda ,\frac{1}{2}}\left(\sigma \right)={\left(h_{\lambda ,\frac{1}{2}}\left(\sigma_{1}\right),h_{\lambda ,\frac{1}{2}}\left(\sigma_{2}\right)...,h_{\lambda ,\frac{1}{2}}\left(\sigma_{n_{min}}\right)\right)}^{T_{h}}$, where $T_{h}$ denotes the threshold value [39,43]. After repeating the process for all frontal slices of $\hat{\Sigma }$, the solution for $l_{\frac{1}{2}}$ induced TNN is obtained. Furthermore, the solution’s detailed procedure is summarized and can be found in Algorithm 1.

In real-life contexts, imperfections in an image may occur in different circumstances, such as during acquisition, from any of the display systems or due to the constraints of both material and technological resources [44]. In any of the ways, the presence of noise in the data may adversely affect the outcomes of the algorithms [45]. The accuracy of the clustering algorithms could be improved if the data become noise-free. To meet this objective, each image is extracted by twisting the data tensor, $X\in R^{n_{1}\times N\times n_{3}}$ developed for the clustering model, and $\vec{X}\in R^{n_{1}\times n_{3}\times N}$ return the twisted tensor [46]. The $k^{th}$ image is then transformed into the $k^{th}$ frontal slice, ${\vec{X}}^{\left(k\right)}\in R^{n_{1}\times n_{3}}$ of the twisted tensor, $\vec{X}$, where k=1 to *N*. This further allows each individual image to be taken in sequence by calling $\vec{X}\left(:,:,i\right)\in R^{n_{1}\times n_{3}}$, where i=1 to *N*. The removal of noise from an image can be achieved by the sparse and low rank matrix decomposition method and is already illustrated in Section 2.1. The concept of removing noise from a single image is given in Equation (3). Then, for *N* number of images, Equation (3) can be modified such that

(11) $$\begin{array}{cc} \min_{T,S}\; & \sum_{k=1}^{N}{∥T^{\left(k\right)}∥}_{*}+\sum_{k=1}^{N}{∥S^{\left(k\right)}∥}_{1}+\frac{1}{2}\sum_{k=1}^{N}{∥{\vec{X}}^{\left(k\right)}-T^{\left(k\right)}-S^{\left(k\right)}∥}_{F}^{2} \end{array}$$

To incorporate all the challenges aforementioned, proposed method integrates the following aspects into its optimization problem.

1. Compared to TNN, $l_{\frac{1}{2}}$ induced TNN is able to capture better low rankness. In addition, due to its inherent noise robustness and better ability to catch the property of self-expressiveness, $l_{\frac{1}{2}}$ induced TNN is introduced into the proposed method.
2. Compared to $l_{1}$ norm, $l_{\frac{1}{2}}$ norm regularization is able to capture the f-block diagonal structure in a better way such that the submodule structure constraint is modified using $l_{\frac{1}{2}}$ norm.
3. To meet the objective of noise removal, we use the tensor $\vec{X}\in R^{n_{1}\times n_{3}\times N}$, where $\vec{X}$ is the twisted version of the noisy data tensor $X\in R^{n_{1}\times N\times n_{3}}$. Afterwards, noise removal is carried out by employing the nuclear norm and $l_{1}$ norm minimization on each image to separate the noise content by combining the principles of sparse and low rank decomposition techniques. As a result, the underlying images are restored, and the sparse noise content is eliminated. This process delivers a noise-free data tensor for further clustering process.

Incorporating the aforementioned factors, the tensor, $T\in R^{n_{1}\times n_{3}\times N}$ is introduced into the proposed optimization problem such that T is the clean data tensor, where the noise removed images are stacked into its frontal slices, $T^{\left(k\right)}\in R^{n_{1}\times n_{3}}$. Another tensor, $S\in R^{n_{1}\times n_{3}\times N}$ is defined, where the eliminated sparse noise content from each image is stored into its frontal slices, $S^{\left(k\right)}\in R^{n_{1}\times n_{3}}$. In addition, the tensor, $R\in R^{n_{1}\times N\times n_{3}}$ is incorporated, where R is the twisted version of the clean data tensor T such that R is given for the clustering. Further, we employ variable splitting for Z into Equation (6) such that Z=C and Z=Q [21]. Combining all the above, the proposed optimization problem can be reformulated as

(12) $$\begin{array}{cc} \min_{C,Q,Z} & \;{∥C∥}_{⊛\frac{1}{2}}+\lambda_{1}\sum_{k=1}^{n_{3}}{∥M⊙Q^{\left(k\right)}∥}_{\frac{1}{2}}^{\frac{1}{2}}+\lambda_{2}{∥R-R*Z∥}_{F}^{2}\\ & +\lambda_{3}\sum_{k=1}^{N}{∥T^{\left(k\right)}∥}_{*}+\lambda_{4}\sum_{k=1}^{N}{∥S^{\left(k\right)}∥}_{1}\\ \; & s.\;t.\;Z=C,\;Z=Q,\;R=\vec{T},\;\sum_{k=1}^{N}\left({\vec{X}}^{\left(k\right)}=T^{\left(k\right)}+S^{\left(k\right)}\right) \end{array}$$

where ${∥.∥}_{⊛\frac{1}{2}}$ represents the $l_{\frac{1}{2}}$ induced TNN and ${∥.∥}_{\frac{1}{2}}^{\frac{1}{2}}$ represents $l_{\frac{1}{2}}$ norm. Further, ${∥.∥}_{*}$, ${∥.∥}_{1}$ and ${∥.∥}_{F}$ denote the nuclear norm, $l_{1}$ norm and Frobenius norm respectively. Finally, $\vec{X}$ is the twisted version of the noisy data tensor $X\in R^{n_{1}\times N\times n_{3}}$. In the above expression, $\lambda_{1}$, $\lambda_{2}$, $\lambda_{3}$ and $\lambda_{4}$ denote the regularization parameters of the proposed optimization problem and among them, $\lambda_{3}$ and $\lambda_{4}$ balance the effect of low rank and sparsity constraints [27]. The above constrained equation is transformed into a unconstrained one using the Augmented Lagrangian (AL) method [19,47] given by

(13) $$\begin{array}{cc} L(C, & Q,Z,G_{1},G_{2},G_{3},G_{4})={∥C∥}_{⊛\frac{1}{2}}+\lambda_{1}\sum_{k=1}^{n_{3}}{∥M⊙Q^{\left(k\right)}∥}_{\frac{1}{2}}^{\frac{1}{2}}\\ & +\lambda_{2}{∥R-R*Z∥}_{F}^{2}+\lambda_{3}\sum_{k=1}^{N}{∥T^{\left(k\right)}∥}_{*}+\lambda_{4}\sum_{k=1}^{N}{∥S^{\left(k\right)}∥}_{1}\\ & +\langle G_{1},Z-C\rangle +\langle G_{2},Z-Q\rangle +\langle G_{3},R-\vec{T}\rangle +\langle G_{4},{\vec{X}}^{\left(k\right)}-T^{\left(k\right)}-S^{\left(k\right)}\rangle \\ & +\frac{\mu }{2}\left({∥Z-C∥}_{F}^{2}+{∥Z-Q∥}_{F}^{2}+{∥R-\vec{T}∥}_{F}^{2}+\sum_{k=1}^{N}{∥{\vec{X}}^{\left(k\right)}-T^{\left(k\right)}-S^{\left(k\right)}∥}_{F}^{2}\right) \end{array}$$

where the tensors $G_{1}$, $G_{2}$, $G_{3}$ and $G_{4}$ are the Lagrangian multipliers, where μ≥0 is the penalty parameter and 〈.,.〉 denotes the inner product [27]. The above problem can be solved by iteratively minimizing the Lagrangian L over one tensor while keeping the others constant [6].

C Subproblem: The update expression for C is given by

(14) $$\begin{array}{c} C^{\left[j+1\right]}=\underset{C}{\arg\;\min}\;{∥C∥}_{⊛\frac{1}{2}}+\langle G_{1},Z-C\rangle +\frac{\mu }{2}{∥Z-C∥}_{F}^{2} \end{array}$$

The above expression can be transformed into the following form,

(15) $$C^{\left[j+1\right]}=\underset{C}{\arg\;\min}\;{∥C∥}_{⊛\frac{1}{2}}+\frac{\mu^{\left[j\right]}}{2}{∥C-\left(Z^{\left[j\right]}-\frac{G_{1}^{\left[j\right]}}{\mu^{\left[j\right]}}\right)∥}_{F}^{2}$$

Solution to the above subproblem is obtained by,

(16) $$\;\;\;\;\;\;\;\;\;\;\;\;\;\;C^{\left[j+1\right]}=H_{\tau }\left[Z^{\left[j\right]}-\frac{G_{1}^{\left[j\right]}}{\mu^{\left[j\right]}}\right]$$

where $\tau =\frac{1}{\mu }$ is the threshold value. The operation of $H_{\tau }\left[.\right]$ is detailed in Algorithm 1.
**Algorithm 1** Tensor singular value half thresholding.**Require:**$Z\in R^{N\times N\times n_{3}},\lambda >0,\mu >0$, threshold, $T_{h}>0$**Ensure:** Singular Value Half-thresholded, $Z_{ht}\in R^{N\times N\times n_{3}}$ as optimal solution1: $\hat{Z}=\mathrm{fft}\left(Z,3\right)$2: **for** i= 1 to $n_{3}$ **do**3:    $\left[U,\Sigma ,V\right]=\mathrm{svd}\left({\hat{Z}}^{\left(i\right)}\right)$4:    ${\hat{U}}^{\left(i\right)}=U,{\hat{\Sigma }}^{\left(i\right)}=\Sigma ,{\hat{V}}^{\left(i\right)}=V$5:    $\sigma =\mathrm{diag}({\hat{\Sigma }}^{\left(i\right)})$6:    $H_{\lambda ,\frac{1}{2}}\left(\sigma \right)={\left(h_{\lambda ,\frac{1}{2}}\left(\sigma_{1}\right),h_{\lambda ,\frac{1}{2}}\left(\sigma_{2}\right),\ldots h_{\lambda ,\frac{1}{2}}\left(\sigma_{N}\right)\right)}^{T_{h}}$7:    ${\hat{\Sigma }}_{hf}\left(:,:,i\right)=\mathrm{diag}\left(H_{\lambda ,\frac{1}{2}}\left(\sigma \right)\right)$8: **end for**9: $U=\mathrm{ifft}(\hat{U},3)$, $H_{\frac{1}{2}}\left(\Sigma_{t}\right)=\mathrm{ifft}\left({\hat{\Sigma }}_{hf},3\right)$, $V=\mathrm{ifft}(\hat{V},3)$10: $Z_{ht}=U*H_{\frac{1}{2}}\left(\Sigma_{t}\right)*V^{T}$

Q Subproblem: The update expression for Q is given by

(17) $$\begin{array}{cc} Q^{\left[j+1\right]}= & \underset{Q}{\arg\;\min}\;\lambda_{1}\sum_{k=1}^{n_{3}}{∥M⊙Q^{\left(k\right)}∥}_{\frac{1}{2}}^{\frac{1}{2}}+\langle G_{2},Z-Q\rangle +\frac{\mu }{2}{∥Z-Q∥}_{F}^{2} \end{array}$$

Above equation can be decomposed into $n_{3}$ expressions and the *kth* frontal slice of Q can be updated by

(18) $$\begin{array}{cc} Q^{{\left(k\right)}^{\left[j+1\right]}}= & \underset{Q}{\arg\;\min}\;\lambda_{1}{∥M⊙Q^{\left(k\right)}∥}_{\frac{1}{2}}^{\frac{1}{2}}+\frac{\mu^{\left[j\right]}}{2}{∥Q-\left(Z^{{\left(k\right)}^{\left[j\right]}}+\frac{G_{2}^{{\left(k\right)}^{\left[j\right]}}}{\mu^{\left[j\right]}}\right)∥}_{F}^{2} \end{array}$$

where $Q^{{\left(k\right)}^{\left[j+1\right]}}$ is the $k^{th}$ frontal slice/matrix of Q. The solution to the above subproblem is given by the halfthresholding operator [42],

(19) $$\begin{array}{c} \;\;\;\;\;\;\;\;Q^{{\left(k\right)}^{\left[j+1\right]}}=H_{\frac{\lambda_{1}M}{\mu^{i}}}\left[Z^{{\left(k\right)}^{\left[j\right]}}+\frac{G_{2}^{{\left(k\right)}^{\left[j\right]}}}{\mu^{\left[j\right]}}\right] \end{array}$$

where $H_{\frac{\lambda_{1}M}{\mu^{i}}}$ is the halfthresholding operator [39]. Here, $Q_{m,n}^{\left(k\right)}$ is the (m,n)*th* element of *kth* frontal slice/matrix of Q.

Z Subproblem: The subproblem for updating Z is given by

(20) $$\begin{array}{cc} Z^{\left[j+1\right]}=\underset{Z}{\arg\;\min}\; & \lambda_{2}{∥R-R*Z∥}_{F}^{2}+\langle G_{1}^{\left[j\right]},Z-C^{\left[j+1\right]}\rangle +\frac{\mu^{\left[j\right]}}{2}{∥Z-C^{\left[j+1\right]}∥}_{F}^{2}\\ & +\frac{\mu^{\left[j\right]}}{2}{∥Z-Q^{\left[j+1\right]}∥}_{F}^{2}+\langle G_{2}^{\left[j\right]},Z-Q^{\left[j+1\right]}\rangle \end{array}$$

Above equation can be simplified as,

(21) $$\begin{array}{cc} Z^{\left[j+1\right]} & =\underset{Z}{\arg\;\min}\;\lambda_{2}{∥R-R*Z∥}_{F}^{2}+\frac{\mu^{\left[j\right]}}{2}\left({∥Z-C^{\left[j+1\right]}∥}_{F}^{2}+{∥Z-Q^{\left[j+1\right]}∥}_{F}^{2}\right) \end{array}$$

Finding the Fourier transform on both sides, the above equation can be rewritten as,

(22) $$\begin{array}{c} {\hat{Z}}^{\left[j+1\right]}=\underset{\hat{Z}}{\arg\;\min}\;\lambda_{2}{∥\hat{R}-\hat{R}⊗\hat{Z}∥}_{F}^{2}+\frac{\mu^{\left[j\right]}}{2}\left({∥\hat{Z}-{\hat{P}}_{1}^{\left[j+1\right]}∥}_{F}^{2}+{∥\hat{Z}-{\hat{P}}_{2}^{\left[j+1\right]}∥}_{F}^{2}\right) \end{array}$$

where $\hat{Z}$, ${\hat{P}}_{1}^{\left[j+1\right]}$ and ${\hat{P}}_{2}^{\left[j+1\right]}$ are the Fourier transforms of *kth* frontal slice of Z, $C^{\left[j+1\right]}-\frac{G_{1}^{\left[j\right]}}{\mu^{\left[j\right]}}$ and $Q^{\left[j+1\right]}-\frac{G_{2}^{\left[j\right]}}{\mu^{\left[j\right]}}$, respectively, and ${}^{‵}{⊗}^{\prime }$ indicates the slicewise multiplication [19]. The analytic solution for the update of the *kth* frontal slice is given by

(23) $$\begin{array}{c} {\hat{Z}}^{{\left(k\right)}^{\left[j+1\right]}}=\left(2\lambda_{2}{\hat{R}}^{{\left(k\right)}^{T}}{\hat{R}}^{\left(k\right)}+\mu^{\left[j\right]}\left({\hat{P}}_{1}^{{\left(k\right)}^{\left[j+1\right]}}+{\hat{P}}_{2}^{{\left(k\right)}^{\left[j+1\right]}}\right)\right){\left(2\lambda_{2}{\hat{R}}^{{\left(k\right)}^{T}}{\hat{R}}^{\left(k\right)}+2\mu^{\left[j\right]}I\right)}^{-1} \end{array}$$

T Subproblem: In T subproblem, the update expression is given by,

(24) $$\begin{array}{cc} T^{\left[j+1\right]} & =\underset{T}{\arg\;\min}\;\lambda_{3}\sum_{k=1}^{N}{∥T^{\left(k\right)}∥}_{*}+\langle G_{4},{\vec{X}}^{\left(k\right)}-T^{\left(k\right)}-S^{\left(k\right)}\rangle +\frac{\mu }{2}{∥{\vec{X}}^{\left(k\right)}-T^{\left(k\right)}-S^{\left(k\right)}∥}_{F}^{2} \end{array}$$

Above expression can be considered an *N* subproblems. Then, the update expression for *kth* slice is given by,

(25) $$\begin{array}{cc} \; & T^{{\left(k\right)}^{\left[j+1\right]}}=\underset{T}{\arg\;\min}\;\lambda_{3}\sum_{i=1}^{N}{∥T^{{\left(k\right)}^{\left[j\right]}}∥}_{*}+\mu^{\left[j\right]}\left(∥T^{{\left(k\right)}^{\left[j\right]}}-\left({\vec{X}}^{{\left(k\right)}^{\left[j\right]}}-S^{{\left(k\right)}^{\left[j\right]}}+\frac{G_{4}^{{\left(k\right)}^{\left[j\right]}}}{\mu^{\left[j\right]}}\right)∥\right) \end{array}$$

Above expression can be solved using singular value thresholding,

(26) $$\begin{array}{c} T^{{\left(k\right)}^{\left[j+1\right]}}=S_{\frac{\lambda_{3}}{\mu^{\left[j\right]}}}\left[{\vec{X}}^{{\left(k\right)}^{\left[j\right]}}-S^{{\left(k\right)}^{\left[j\right]}}+\frac{G_{4}^{{\left(k\right)}^{\left[j\right]}}}{\mu^{\left[j\right]}}\right] \end{array}$$

$S_{\frac{\lambda_{3}}{\mu^{\left[j\right]}}}\left[.\right]$ is the singular value thresholding operator [48].

S Subproblem: Similarly, the update expression for S subproblem is given by

(27) $$\begin{array}{cc} S^{\left[j+1\right]} & =\underset{T}{\arg\;\min}\;\lambda_{4}\sum_{i=1}^{N}{∥S^{\left(k\right)}∥}_{1}+\langle G_{4},{\vec{X}}^{\left(k\right)}-T^{\left(k\right)}-S^{\left(k\right)}\rangle +\frac{\mu }{2}{∥{\vec{X}}^{\left(k\right)}-T^{\left(k\right)}-S^{\left(k\right)}∥}_{F}^{2} \end{array}$$

Solution for the *kth* slice is given by,

(28) $$\begin{array}{c} \;\;\;\;\;\;\;\;S^{{\left(k\right)}^{\left[j+1\right]}}=s_{\frac{\lambda_{4}}{\mu^{\left[j\right]}}}\left[{\vec{X}}^{{\left(k\right)}^{\left[j\right]}}-T^{{\left(k\right)}^{\left[j+1\right]}}+\frac{G_{4}^{{\left(k\right)}^{\left[j\right]}}}{\mu^{\left[j\right]}}\right] \end{array}$$

where $s_{\frac{\lambda_{4}}{\mu^{\left[j\right]}}}\left[.\right]$ is the shrinkage operator defined in [27] and the expression is given by, $s_{\theta >0}\left(x\right)=sign\left(x\right)max\left(|x|-\theta ,0\right)$, where θ represents the threshold value.

R Subproblem:

(29) $$\begin{array}{cc} R^{\left[j+1\right]} & =\underset{R}{\arg\;\min}\;\lambda_{2}{∥R-R*Z∥}_{F}^{2}+\langle G_{3},R-\vec{T}\rangle +\frac{\mu }{2}{∥R-\vec{T}∥}_{F}^{2} \end{array}$$

Solution for the above expression is given by,

(30) $$\begin{array}{cc} R^{\left[j+1\right]} & ={\left(2\lambda_{2}Z^{\left[j+1\right]}-2\lambda_{2}Z^{\left[j+1\right]}\times Z^{T^{\left[j+1\right]}}-\mu^{\left[j\right]}I\right)}^{-1}\left(G_{3}^{\left[j\right]}+\mu^{\left[j\right]}{\vec{T}}^{\left[j+1\right]}\right) \end{array}$$

Finally, the stopping criterion is measured by the following condition,

(31) $$\begin{array}{c} max\left\{\begin{array}{ccc} {∥Z^{\left[j+1\right]}-C^{\left[j\right]}∥}_{\infty }, & {∥Z^{\left[j+1\right]}-Q^{\left[j\right]}∥}_{\infty }, & {∥Z^{\left[j+1\right]}-Z^{\left[j\right]}∥}_{\infty }\\ {∥C^{\left[j+1\right]}-C^{\left[j\right]}∥}_{\infty }, & {∥Q^{\left[j+1\right]}-Q^{\left[j\right]}∥}_{\infty }, & {∥T^{\left[j+1\right]}-T^{\left[j\right]}∥}_{\infty } \end{array}\right\}<\epsilon \end{array}$$

The overall algorithm can be summarized in Algorithm 2.
**Algorithm 2** Robust tensor-based submodule clustering for noisy imaging data.**Require:** Data: $X\in R^{n_{1}\times N\times n_{3}}$ and parameters $\lambda_{1}$, $\lambda_{2}$, $\lambda_{3}$, $\lambda_{4}$, $\mu_{max}$, ρ**Ensure**: $Z\in R^{N\times N\times n_{3}}$, $T\in R^{n_{1}\times n_{3}\times N}$1: $C^{\left[0\right]}$=$Q^{\left[0\right]}$=$Z^{\left[0\right]}$=$G_{1}^{\left[0\right]}$=$G_{2}^{\left[0\right]}$$\leftarrow 0\in R^{N\times N\times n_{3}}$2: $T^{\left[0\right]}$=$S^{\left[0\right]}$=$G_{4}^{\left[0\right]}\leftarrow 0\in R^{n_{1}\times n_{3}\times N}$3: $R^{\left[0\right]}$=$G_{3}^{\left[0\right]}\leftarrow 0\in R^{n_{1}\times N\times n_{3}}$ and t←04: $\lambda_{1}>0$, $\lambda_{2}>0$, $\lambda_{3}>0$, $\lambda_{4}>0$, $\mu^{\left[0\right]}>0$, ρ>05: **while** not converged **do**6:   $C^{\left[j+1\right]}$← Update using Equation (16)7:   $Q^{\left[j+1\right]}$← Update using Equation (18)8:   $Z^{\left[j+1\right]}$← Update using Equation (23)9:   $T^{\left[j+1\right]}$← Update using Equation (24)10:   $S^{\left[j+1\right]}$← Update using Equation (28)11:   $R^{\left[j+1\right]}$← Update using Equation (30)12:   $G_{1}^{\left[j+1\right]}$=$G_{1}^{\left[j\right]}+\mu^{\left[j\right]}\left(Z-C\right)$13:   $G_{2}^{\left[j+1\right]}$=$G_{2}^{\left[j\right]}+\mu^{\left[j\right]}\left(Z-Q\right)$14:   $G_{3}^{\left[j+1\right]}$=$G_{3}^{\left[j\right]}+\mu^{\left[j\right]}\left(R-\vec{T}\right)$15:   $G_{4}^{\left[j+1\right]}$=$G_{4}^{\left[j\right]}+\mu^{\left[j\right]}\sum_{k=1}^{N}\left(X^{\left(k\right)}-T^{\left(k\right)}-S^{\left(k\right)}\right)$16:   $\mu^{\left[j+1\right]}$=$\rho \mu^{\left[j\right]}$17:   Check the convergence using Equation (31)18:   [j]←[j+1]19: **end while**

## 4. Results and Discussions

The performance of the proposed method is evaluated on Coil20 http://www.cs.columbia.edu/CAVE/software/softlib/coil-20.php (accessed on 5 June 2021), MNIST http://yann.lecun.com/exdb/mnist/ (accessed on 5 June 2021) and UCSD http://www.svcl.ucsd.edu/projects/anomaly/dataset.htm (accessed on 8 June 2021) datasets [19]. These datasets are widely used for clustering, completion, noise reduction, and moving object detection problems [6,19,42]. The dimensions, number of classes and total number of images of these datasets have already been defined in various papers and can be found in [6,24] and so on. For comparison with the proposed method, other recent clustering methods, such as normalized spectral clustering [16], SSmC [20], SLRSmC [21], SCLRSmC [19], weighted tensor nuclear norm (WTNN) minimization [23] and re-weighted low rank tensor approximation and $l_{\frac{1}{2}}$ regularization, named $\left(\mathrm{RLRTA}l_{\frac{1}{2}}R\right)$[24], approaches are chosen. All the experiments are implemented and run on a personal computer with i5 - 4590 CPU at 3.30 GHz and 8 GB of RAM. The results are compared using the standard evaluation metrics such as the misclustering rate (MCR), adjusted Rand index (ARI), normalized mutual information (NMI) and purity [6]. The definitions and expressions of MCR, ARI and purity can be found in [6,24]. Then, normalized mutual information (NMI) is obtained by normalizing the mutual information to a value between 0 and 1, where the value of 1 indicates perfect labeling. In addition, purity and ARI are upper bound measures whose values lie in the interval (0,1)[24]. For those metrics, higher values indicate sound performance. In this work, the clustering results for MCR are represented by (m±σ)%, where *m* is the mean, σ is the standard deviation and smaller MCR value indicates improved performance [6]. To simulate sparse noise in the data, we create an algorithm which generates noise values at random locations in the images. The amount of sparse noise applied to the data can be modified by this algorithm, and the amount of sparse noise added is shown as a percentage of the total pixels in the images of each dataset. In this work, the amount of sparse noise are varied from 5% to 50% for all the datasets.

**Figure 3:**
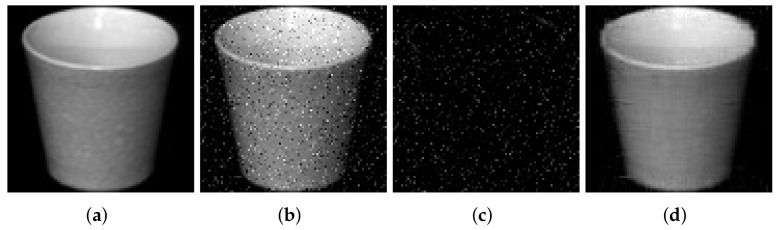
Illustration of noise removal of a single image from Coil20 dataset. (**a**) Input image (**b**) Image with 20% sparse noise (**c**) Sparse noise content (**d**) Noise removed image.

We first present the experimental results obtained from the Coil20 dataset. For the Coil20 dataset, the values chosen for the regularization parameters are $\lambda_{3}=1.155$ and $\lambda_{4}=0.355$. The proposed method exhibits a major improvement in its clustering efficiency, and furthermore improved evaluation metrics are obtained. The reason is that the proposed algorithm decomposes each image in the dataset into its sparse and low rank part. The sparse part represents the noise encountered, and the algorithm removes this sparse noise content. Consequently, the imaging data are free from noise and clean data are available for clustering at the same time. Figure 3 shows the visual appearance of the simultaneous noise removal of a single image from the coil20 dataset with 20% sparse noise applied. The eliminated noise content from the noisy image is presented in Figure 3c, and the recovered clean image is shown in Figure 3d, respectively.

**Figure 4:**
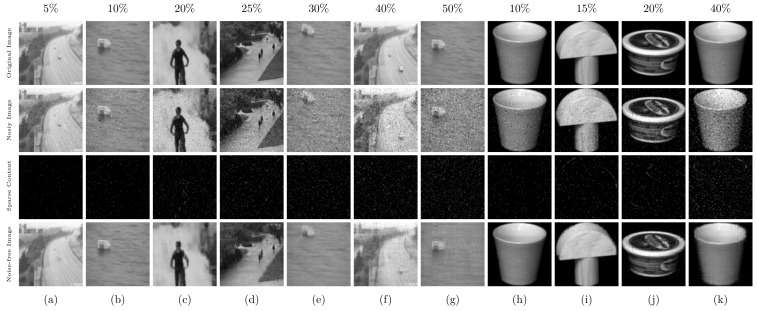
Illustrated noise-removed images achieved using proposed method from UCSD dataset (**a**–**g**) and Coil20 dataset (**h**–**k**) for various levels of sparse noise. The sparse noise levels are indicated on the top of each input image. First row: original input image, second row: images that have been corrupted by various levels of sparse noise, third row: eliminated sparse noise content from each image, fourth row: noise-removed images.

**Figure 5:**
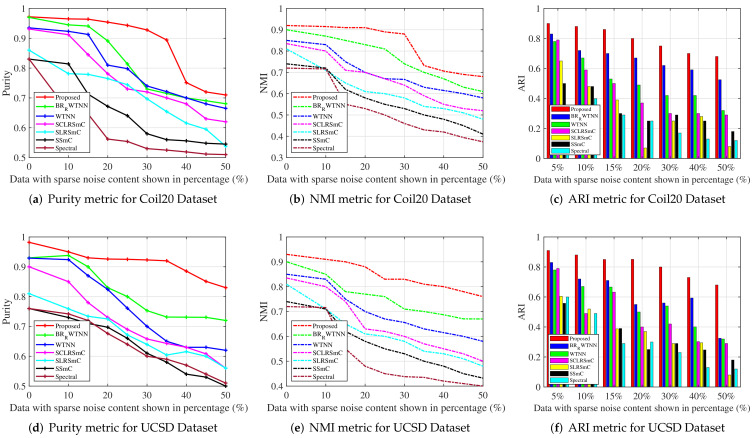
Quantitative comparison of purity, NMI and ARI metrics of the proposed method and state-of-the art algorithms under various levels of sparse noise using Coil20 and UCSD datasets.

The proposed method is compared against state-of-the art clustering algorithms. The MCR values obtained using the Coil20 dataset for proposed method and other algorithms are summarized in the first section of Table 1, with the best values shown in bold. Similarly, the compared results of purity, NMI, and ARI metrics using Coil20 dataset are shown in Figure 5a, Figure 5b and Figure 5c, respectively. Our method obtains better values of MCR, purity, NMI and ARI metrics, compared to the state-of-the-art methods. For 10% to 20% of sparse noise content, the MCR values of the proposed method are (0.67±1.08)% and (2.56±4.42)% (second and third row, last column of Table 1), and these values are extremely small, compared to other algorithms. Similarly, purity and ARI values of the proposed method for 20% of sparse noise content are 0.965, 0.922 and 0.912, respectively (from Figure 5). Hence, it is evident from Table 1 and Figure 5 that the proposed method outperforms other state-of-the-art clustering algorithms. The evaluation metrics of the proposed method indicate small decrements for noise values exceeding 35% and more, but the values are still better than its counterparts. Further, the noise-removed images achieved by the proposed method using the Coil20 dataset for various levels of sparse noise content are shown in Figure 4. The eliminated sparse noise content from the noisy images in the dataset are clearly illustrated in the third row of Figure 4.

Second, experiments are conducted on the UCSD dataset, and the obtained MCR values are provided in the second section of Table 1. For lower noise content ( 5% to 20%), the proposed approach achieves smaller MCR values as reported in Table 1. For noise values such as 30% and 40%, the MCR values for the proposed method are (10.62±9.44)% and (14.05±8.92)%. In the same scenario, algorithms such as SSmC, SLRSmC and SCLRSmC fail to achieve good clustering results (Table 1, Figure 5d–f). WTNN shows improved results over the SSmC, SLRSmC, SCLRSmC and spectral methods for lower noise values, but its performance reduces when the noise content in the imaging data is increased. Among the methods we have compared, the $RLRTAl_{\frac{1}{2}}R$ method shows the second best performance. The MCR metrics of this method are (0.75±1.96)% to (15.33±10.90)% for noise levels up to 30%. In all of the scenarios considered, the proposed approach outperforms the state-of-the-art methods significantly. In addition, $l_{\frac{1}{2}}$ norm regularization effectively captures the f-block diagonal structure in a better way, and the self-expressiveness property of the submodules is preserved in the proposed method. A few images from the UCSD dataset, which was recovered by the proposed method under various noise levels, are shown in Figure 4. The proposed method’s efficiency is also checked using the MNIST dataset [19]. the MNIST dataset comprises images of handwritten digits from 0 to 9 with a resolution of 28×28, where the number of images that belong to class is set as 30. The MCR metrics obtained for the proposed as well as the compared methods are summarized in last section of Table 1. The proposed method produces better clustering results than the state-of-the-art methods.

To summarize, the proposed approach performs well and provides good clustering results, even with noise-corrupted data for these three datasets. Furthermore, it outperforms all of the clustering algorithms that we compare in this work throughout every case. The reasons for the improved performance are as follows: first, the proposed algorithm is a unified optimization framework that clusters imaging data while also eliminating noise from images. Furthermore, $l_{\frac{1}{2}}$ induced TNN, incorporated into the proposed method, provides better low rankness and maintains the self-expressiveness property of submodules. Second, the proposed method’s optimization equation demonstrates the benefit of $l_{\frac{1}{2}}$ regularization in providing better submodule identification. Finally, the simultaneous noise removal reduces the impact of noise on the clustering performance and provides clean data for further clustering. No other methods in the state of the art have a simultaneous noise reduction scheme in their optimization problem for extracting the noise content from individual images. In most of the approaches, a global error term is used in their optimization problems to reduce the effect of noise when clustering. However, it is just a partial solution that cannot be applied to all cases. On the other hand, the proposed method handles individual images in the dataset and removes the noise content simultaneously, which is the major contribution of the proposed method.

### 4.1. Analysis of the Proposed Method with Gaussian Noise and Salt and Pepper Noise

**Figure 6:**
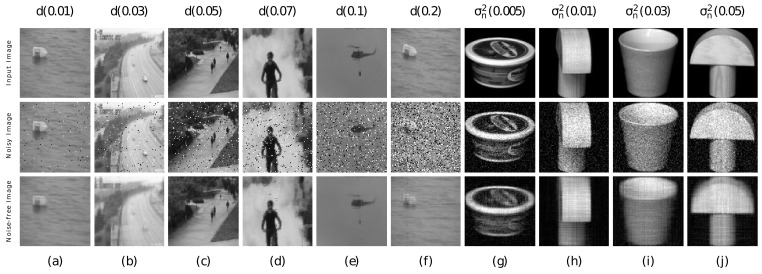
Noise-removed images achieved using proposed method from UCSD and Coil20 datasets for various levels of salt and pepper and Gaussian noise. The noise levels of salt and pepper noise and Gaussian noise are indicated on the top of each input image. First row: original input image, second row: images that have been corrupted by various levels of salt and pepper and Gaussian noise, third row: noise-removed images.

**Figure 7:**
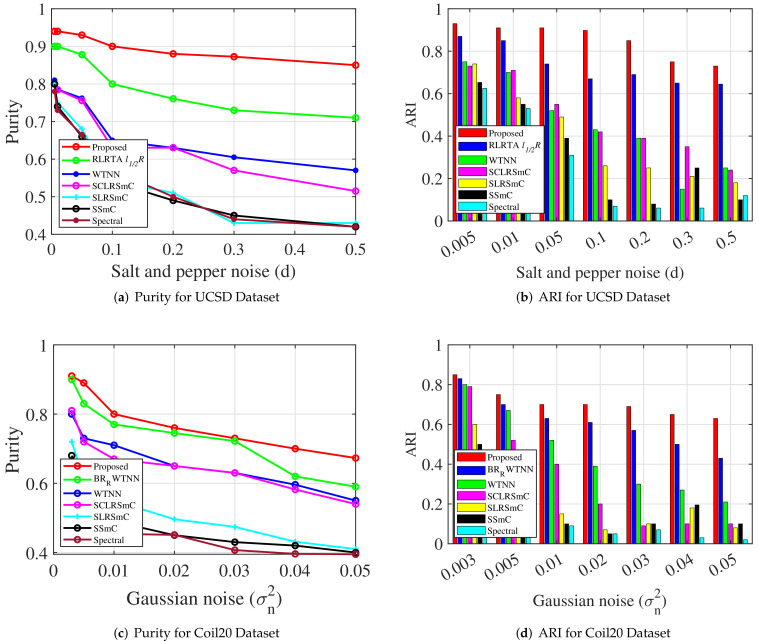
Quantitative Comparison (**a**,**b**): purity metric and ARI metric for UCSD dataset for various levels of salt and pepper noise. (**c**,**d**): purity metric and ARI metric for Coil20 dataset for various levels of Gaussian noise. of purity and ARI metrics of the proposed method and state-of-the art algorithms under salt and pepper noise (*d*) and Gaussian noise ($\sigma_{n}^{2}$).

In order to further analyze the robustness of the proposed system, experiments were conducted on imaging data that were distorted by different types of noise [49,50]. For this study, we used Coil20 and UCSD datasets, and two cases were considered. In case I, images that are corrupted by salt and pepper noise were considered. Salt and pepper noise is a type of impulse noise, where the noise values include two extreme ranges of a pixel value [28]. In one study, an impulse noise removal algorithm based on the sparse and low rank decomposition method was proposed in which impulse noise types were modeled as sparse components and the underlying image was restored with keeping the original features [28]. In this work, salt and pepper noise of various noise densities (*d*) were added. The noise density level considered are d=0.03 to 0.3. In the presence of salt and pepper noise, it was observed that the proposed method provides improved clustering performance as well as restoring the clean image with removed noise content. To substantiate, a few of the recovered images from the UCSD dataset are displayed in Figure 6. In this, the first row represents the original images, the second row denotes the noise corrupted images of various noise densities and the last row represents the recovered images. Further, the MCR metrics under salt and pepper noise are presented in Case I of Table 2. Similarly, purity and ARI metrics of our method and the compared methods for UCSD dataset are shown in Figure 7a and Figure 7b, respectively. In all cases, the proposed method outperforms the state-of-the-art algorithms that we compared.

In case II, images corrupted by Gaussian noise were used. Gaussian noise in images is most common when the lighting is low or the temperature is high [27]. This can happen at any time during the capture or transmission process. In analysis, the noise variances considered are $\sigma_{n}^{2}=0.005$, $\sigma_{n}^{2}=0.01$, $\sigma_{n}^{2}=0.02$, $\sigma_{n}^{2}=0.03$, $\sigma_{n}^{2}=0.05$, $\sigma_{n}^{2}=0.07$ and $\sigma_{n}^{2}=0.1$. Case II in Table 2 summarizes the MCR metrics of the proposed method and the compared methods. Figure 7c,d shows the compared results of purity and ARI metrics, respectively. The obtained images of the proposed method under Gaussian noise are shown in the last four columns of Figure 6. For noise variances up to $\sigma_{n}^{2}=0.03$, our method successfully recovers the noise-free images, but for noise variance values of $\sigma_{n}^{2}=0.05$ or more, the recovered images have an over-smoothing problem. However, by fine-tuning the regularization parameters, this issue can be mitigated to a certain extent. Nonetheless, the compared methods generate inadequate clustering performance under the same scenarios. The second-best performance is achieved by the $RLRTAl_{\frac{1}{2}}R$ approach. Then, WTNN performs reasonably to an extent for lower noise variances, but the results are deteriorated for higher noise values. In comparison to the above methods, the other methods do not perform as well. Hence, in the presence of Gaussian noise at different noise levels, the proposed method performs well and outperforms state-of-the-art clustering methods in general.

### 4.2. Parameter Tuning and Convergence Analysis

**Figure 8:**
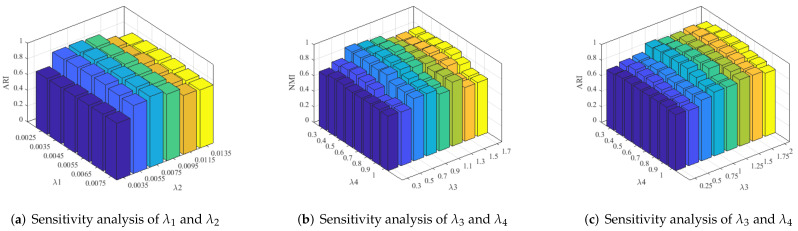
Sensitivity analysis of the proposed method with the evaluation metrics NMI and ARI. (**a**) Sensitivity analysis of $\lambda_{1}$ and $\lambda_{2}$ with ARI metric using Coil20 dataset, (**b**) Sensitivity analysis of $\lambda_{3}$ and $\lambda_{4}$ with NMI metric using Coil20 dataset. (**c**) Sensitivity analysis of $\lambda_{3}$ and $\lambda_{4}$ with ARI metric using UCSD dataset.

The sensitivity analysis of all regularization parameters against the evolution metrics NMI and ARI, and the convergence of the proposed algorithm are discussed in this section. The parameters $\lambda_{3}$ and $\lambda_{4}$ are tuned manually to obtain the best results from the range of (0.2–2.1). Figure 8b,c illustrates graphs of the two metrics, NMI and ARI, with these regularization parameters. The graphs show that the proposed method provides good evaluation scores for $\lambda_{3}$ within the range of 0.70–1.5 and $\lambda_{4}$ in 0.25–0.95. The optimal values for the proposed method are found to be $\lambda_{3}=1.15$ and $\lambda_{4}=0.65$ within this range. However, to obtain good evaluation scores and visual quality when using different datasets, minor variations can be allowed in these values. Similarly, the parameters $\lambda_{1}$ and $\lambda_{2}$ balance the effect the submodule structure constraint term and representation error term, respectively. The optimal values for $\lambda_{1}$ and $\lambda_{2}$ are identified as $\lambda_{1}=4.5\times 10^{-3}$ and $\lambda_{2}=7.5\times 10^{-3}$. The sensitivity analysis of $\lambda_{1}$ and $\lambda_{2}$ with respect to the ARI metric is shown in Figure 8a.

**Figure 9:**
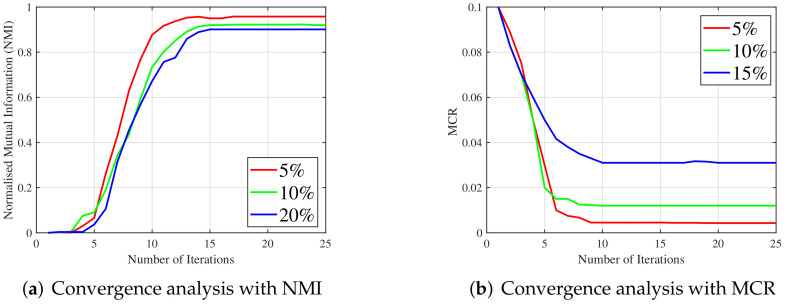
Convergence analysis of the proposed method. (**a**) Convergence analysis of the proposed method with NMI metric using UCSD dataset, (**b**) Convergence analysis of the proposed method with MCR (*m*) metric using Coil20 dataset.

Similarly, the proposed algorithm has a high convergence rate and converges quickly within 10–20 iterations. The proposed method’s convergence curves with the metrics NMI and MCR (with mean value *m*) are plotted in Figure 9a,b, respectively. The plots show that as the number of iterations increases, the change in NMI and MCR values converges to zero. In addition, Table 3 shows the execution time required for the proposed method, compared to the existing methods. Since the proposed algorithm employs $l_{\frac{1}{2}}$ regularization and $l_{\frac{1}{2}}$ induced TNN, their solutions are to be computed iteratively. Furthermore, the proposed method employs nuclear and $l_{1}$ norm minimization for the simultaneous noise removal of every image in the dataset. Therefore, an additional reasonable amount of time is consumed by the proposed method. This marginal increase in computational time can, however, be offset by the use of high-performance computing stations. In addition, the proposed method has six subproblems and four multipliers to update, as presented in Algorithm 2. In the proposed method, $l_{\frac{1}{2}}$ induced TNN, nuclear and $l_{1}$ norm minimization need more computational requirements. $T\in R^{n_{1}\times n_{3}\times N}$ update involves nuclear norm minimization on each slice, which requires $O(\frac{1}{2}Nn_{1}n_{3}^{2})$ operations. In S update, where $S\in R^{n_{1}\times n_{3}\times N}$, it requires $O(Nn_{1}n_{3})$ operations. Finally, $C\in R^{N\times N\times n_{3}}$ update requires $O(N^{2}n_{3}log_{2}n_{3}+\frac{1}{2}n_{3}N^{2})$ operations. Therefore, the total computational complexity of the proposed method is given by $O(T\left(N^{2}n_{3}log_{2}n_{3}+\frac{1}{2}n_{3}N^{2}+\frac{1}{2}Nn_{1}n_{3}^{2}+Nn_{1}n_{3}\right))$ operations, where *T* represents the number of iterations. Hence, the proposed method offers moderate computational complexity.

## 5. Conclusions

This paper proposes a robust tensor-based low rank submodule clustering technique for 2D imaging data with enhanced clustering capability. Traditional clustering methods treat images as vectors, but the proposed method treats them as lateral slices of a third order tensor, which aids in preserving the spatial information of the imaging data. The proposed optimization problem incorporates $l_{\frac{1}{2}}$ induced TNN and $l_{\frac{1}{2}}$ regularization, which facilitates achieving a more accurate low tensor rank approximation and submodule segmentation. Unlike existing clustering techniques, the proposed method incorporates a simultaneous noise reduction scheme by applying the principles of sparse and low rank decomposition techniques to each individual noise corrupted image in the dataset. Afterwards, the noise content is removed, and the underlying clean images are provided for further clustering. The proposed method is compared to state-of-the-art clustering algorithms. The experimental results show that the proposed method outperforms existing state-of-the-art clustering algorithms in terms of NMI, MCR and purity metrics.

## Figures and Tables

**Table 1:** Table represents compared results of MCR (m±σ)% for Coil20, UCSD and MNIST datasets. Best values are highlighted in bold letters.

Dataset	No. of Clusters	Saprse Noise (%)	Spectral [16]	SSmC [20]	SLRSmC [21]	SCLRSmC [19]	WTNN [23]	$RLRTAl_{\frac{1}{2}}R$ [24]	Proposed
Coil 20	3	5	3.95 ± 5.05	6.55 ± 9.66	7.69 ± 4.44	3.13 ± 2.22	1.82 ± 2.12	0.75 ± 1.96	**0.45± 1.14**
		10	9.23 ± 10.53	10 ± 17.23	5.55 ± 9.42	5.05 ± 6.56	5.05 ± 5.25	3.26 ± 1.55	**0.67 ± 1.08**
		15	16.52 ± 14.44	17.32 ± 12.63	9.65 ± 7.48	9.95 ± 4.46	5.95 ± 3.22	7.25 ± 4.62	**2.15 ± 2.90**
		20	21.23 ± 10.12	20.32 ± 10.55	15.55 ± 13.26	10.36 ± 8.05	9.56 ± 7.25	9.05 ± 8.02	**2.56 ± 4.42**
		30	27.32 ± 25.02	26.32 ± 18.99	22.42 ± 17.54	19.25 ± 12.33	17.65 ± 14.22	15.33 ± 10.90	**10.24 ± 11.23**
		40	34.56 ± 16.45	32.99 ± 12.56	33.21 ± 18.10	23.95 ± 16.52	20.44 ± 17.44	18.24 ± 14.64	**14.98 ± 10.15**
		50	38.85 ± 23.23	37.36 ± 20.33	37.77 ± 10.18	28.66 ± 16.12	28.12 ± 19.31	21.02 ± 12.75	**18.44 ± 11.22**
UCSD	3	5	4.04 ± 5.62	7.78 ± 13.47	3.33 ± 4.56	4.12 ± 6.52	5.25 ± 2.12	0.55 ± 1.22	**0.22 ± 0.95**
		10	6.26 ± 9.33	9.97 ± 15.39	4.04 ± 5.09	4.96 ± 5.34	4.80 ± 6.23	1.65 ± 2.32	**1.22 ± 2.54**
		15	14.52 ± 12.22	12.22 ± 21.16	8.4 ± 2.19	6.22 ± 8.92	5.23 ± 6.55	2.23 ± 3.85	**3.04 ± 3.46**
		20	19.52 ± 15.35	16.66 ± 25.86	14.4 ± 6.55	12.27 ± 13.46	11.02 ± 10.52	6.65 ± 5.98	**5.25 ± 3.90**
		30	24.52 ± 12.85	17.66 ± 28.8	18.55 ± 22.19	16.35 ± 15.52	12.52 ± 12.05	11.24 ± 9.25	**10.62 ± 9.44**
		40	31.23 ± 26.22	20.00 ± 26.45	30.05 ± 11.55	14.44 ± 25.01	19.25 ± 20.01	17.40 ± 10.53	**14.05 ± 8.92**
		50	36.23 ± 21.22	38.44 ± 22.19	32.21 ± 10.25	27.45 ± 18.25	27.25 ± 12.22	23.22 ± 6.52	**16.33 ± 7.58**
MNIST	3	5	7.25 ± 2.15	7.15 ± 7.71	7.51 ± 7.22	5.90 ± 5.34	5.55 ± 1.98	2.6 ± 1.35	**1.49 ± 0.95**
		10	9.35 ± 6.35	12.14 ± 11.09	10.05 ± 8.09	9.25 ± 2.94	9.12 ± 2.55	7.65 ± 5.04	**4.40 ± 3.11**
		15	20.04 ± 9.45	17.35 ± 10.12	12.05 ± 9.02	12.43 ± 5.32	10.02 ± 5.37	9.56 ± 7.85	**7.12 ± 4.67**
		20	27.46 ± 9.14	21.56 ± 10.43	27.21 ± 9.12	19.56 ± 12.21	14.29 ± 7.34	11.25 ± 6.04	**7.85 ± 5.45**
		30	25.92 ± 16.05	25.00 ± 12.36	21.73 ± 12.58	22.93 ± 8.46	19.23 ± 7.37	18.63 ± 8.98	**15.22 ± 10.33**
		40	43.86 ± 13.71	26.80 ± 19.55	38.09 ± 9.25	29.19 ± 8.65	29.23 ± 11.45	25.52 ± 13.14	**21.35 ± 10.25**
		50	44.14 ± 17.39	46.83 ± 20.24	45.50 ± 10.21	35.20 ± 15.67	35.45 ± 10.65	31.25 ± 9.58	**28.45 ± 12.45**

**Table 2:** Compared results of MCR (m±σ)% metrics using Coil20 and UCSD datasets under various levels of salt and pepper and Gaussian noise. For salt and pepper noise, noise density *d* and for Gaussian noise, noise variance $\sigma_{n}^{2}$ are added. Best values are highlighted in bold letters.

	Noise Levels	Spectral [16]	SSmC [20]	SLRSmC [21]	SCLRSmC [19]	WTNN [23]	$RLRTAl_{\frac{1}{2}}R$ [24]	Proposed
Case I: Salt and Pepper Noise (*d*)
UCSD	0.03	4.07 ± 5.25	3.95 ± 1.95	3.25 ± 1.04	2.53 ± 1.32	2.04 ± 1.66	1.95 ± 1.45	**0.55 ± 0.64**
	0.05	4.20 ± 7.33	4.41 ± 2.36	3.50 ± 1.68	3.85 ± 1.27	2.32 ± 2.5	2.45 ± 1.80	**0.60 ± 1.15**
	0.1	7.88 ± 5.60	8.45 ± 3.66	5.50 ± 7.22	6.55 ± 4.85	5.25 ± 7.05	3.05 ± 2.45	**1.11 ± 1.80**
	0.2	11.32 ± 4.25	10.50 ± 7.45	11.25 ± 9.38	9.28 ± 6.68	5.38 ± 2.16	6.20 ± 2.56	**1.60 ± 1.55**
	0.3	13.20 ± 7.85	11.75 ± 8.29	31.25 ± 8.67	12.12 ± 7.46	9.05 ± 10.12	8.75 ± 9.55	**3.02 ± 4.54**
Case II: Gaussian Noise $\left(\sigma_{n}^{2}\right)$
Coil20	0.005	3.11 ± 2.42	2.25 ± 3.56	3.04 ± 1.80	2.75 ± 1.04	2.5 ± 1.02	2.5 ± 1.35	**1.13 ± 0.95**
	0.01	9.85 ± 2.85	3.80 ± 7.25	4.57 ± 7.64	3.56 ± 5.83	3.52 ± 5.56	3.05 ± 4.82	**1.45 ± 2.09**
	0.02	12.52 ± 9.50	13.22 ± 9.42	10.15 ± 10.50	9.22 ± 9.23	9.52 ± 7.45	5.52 ± 8.52	**4.21 ± 6.52**
	0.03	18.47 ± 9.35	18.08 ± 6.24	15.45 ± 9.50	11.05 ± 7.15	10.50 ± 5.53	8.25 ± 6.40	**6.16 ± 5.05**
	0.05	18.03 ± 11.51	15.80 ± 10.65	16.74 ± 14.44	13.80 ± 7.42	12.36 ± 9.27	9.54 ± 9.31	**6.27 ± 5.38**
	0.10	23.03 ± 11.51	21.65 ± 10.11	20.33 ± 12.44	19.21 ± 17.52	15.05 ± 11.22	11.80 ± 10.02	**9.03 ± 8.47**

**Table 3:** Execution time comparison of algorithms.

Execution Time Comparison (Sec)			
	**Coil20**	**MNIST**	**UCSD**
**Spectral** [16]	8.7	6.5	9.1
**SSmC** [20]	9.2	8.4	10.3
**SLRSmC** [21]	14.9	13.7	17.5
**SCLRSmC** [19]	17.8	16.7	21.4
**WTNN** [23]	21.6	19.2	24.3
$\mathrm{RLRTA}l_{\frac{1}{2}}R$ [24]	20.5	14.2	21.8
**Proposed**	26.5	22.1	29.5

## Data Availability

The datasets during the current study are publicly available and can be obtained from the below mentioned public domain resources at: http://www.cs.columbia.edu/CAVE/software/softlib/coil-20.php (accessed on 5 June 2021), http://yann.lecun.com/exdb/mnist/ (accessed on 5 June 2021) and http://www.svcl.ucsd.edu/projects/anomaly/dataset.htm (accessed on 8 June 2021).
